# Spindle Cell (Metaplastic) Carcinoma of the Breast Showing No Clinical Benefit with Pembrolizumab-Based Neoadjuvant Chemoimmunotherapy

**DOI:** 10.70352/scrj.cr.26-0071

**Published:** 2026-06-16

**Authors:** Rin Yamada, Keita Kai, Kaori Hidaka, Daiki Yoshii, Ren Shirotani, Tomoka Takahashi, Yuko Miyasato, Seiichi Murakami, Yoshiki Mikami, Yoshihiro Komohara, Yutaka Yamamoto

**Affiliations:** 1 Department of Cell Pathology, Graduate School of Medical Sciences, Kumamoto University, Kumamoto, Kumamoto, Japan; 2 Department of Diagnostic Pathology, Kumamoto University Hospital, Kumamoto, Kumamoto, Japan; 3 Department of Breast and Endocrine Surgery, Kumamoto University Hospital, Kumamoto, Kumamoto, Japan; 4 Department of Surgery, Japan Community Healthcare Organization Amakusa Central General Hospital, Amakusa, Kumamoto, Japan

**Keywords:** breast, metaplastic carcinoma, spindle cell carcinoma, immune checkpoint inhibitor, human leukocyte antigen, β2-microglobulin, tumor-associated macrophage

## Abstract

**INTRODUCTION:**

Metaplastic carcinoma (MC) of the breast is a rare subtype that is frequently chemoresistant, and clinical evidence for immune checkpoint inhibitor (ICI) therapy remains limited. Here, we report a case of spindle cell carcinoma (SpCC) MC showing primary resistance to neoadjuvant chemo-immunotherapy and describe features associated with the tumor immune microenvironment.

**CASE PRESENTATION:**

A woman with a 10-year history of a left breast mass presented with rapid enlargement to approximately 7 cm. Core needle biopsy revealed SpCC that was negative for estrogen receptor, progesterone receptor, and human epidermal growth factor receptor 2, with a high Ki-67 labeling index. Imaging suggested ipsilateral axillary nodal involvement, and neoadjuvant pembrolizumab combined with platinum/taxane chemotherapy was initiated. However, after 2 cycles, the tumor further enlarged with ulceration and protrusion from the prior biopsy site, consistent with progressive disease, and the patient underwent total mastectomy with axillary dissection. Histologically, the tumor consisted of high-grade atypical spindle cells with necrosis and minimal treatment effect. Immunohistochemistry demonstrated strong expression of programmed death-ligand 1 in both tumor and immune cells and increased CD8+ T-cell infiltration after therapy. By contrast, tumor cells showed significantly downregulated human leukocyte antigen (HLA)-A/B/C and β2-microglobulin, suggesting impaired antigen presentation. Increased CD163/CD204/triggering receptor expressed on myeloid cells 2–positive tumor-associated macrophages and focal transforming growth factor-β expression were also observed.

**CONCLUSIONS:**

These findings suggest that HLA class I downregulation may contribute to ICI resistance.

## Abbreviations

B2M
: β2-microglobulin
CTL
: cytotoxic T lymphocyte
FDG
: fluorodeoxyglucose
HLA
: human leukocyte antigen
ICI
: immune checkpoint inhibitor
IHC
: immunohistochemistry
MC
: metaplastic carcinoma
PD-L1
: programmed death-ligand 1
SpCC
: spindle cell carcinoma
sTIL
: stromal tumor-infiltrating lymphocyte
TAM
: tumor-associated macrophage
TGF-β
: transforming growth factor-β
TNBC
: triple-negative breast cancer
Treg
: regulatory T cell
TREM
: triggering receptor expressed on myeloid

## INTRODUCTION

MC is a rare special type of breast carcinoma that accounts for less than 1% of all invasive breast carcinomas.^1)^ It is characterized by squamous, spindle, and/or mesenchymal differentiation and, according to the direction of differentiation, is classified into several histologic patterns—low-grade adenosquamous carcinoma, fibromatosis-like MC, SpCC, squamous cell carcinoma, and MC with heterologous mesenchymal differentiation—as defined in the 5th edition of the World Health Organization Classification of Tumours.^2)^ SpCC is characterized by the proliferation of atypical spindle cells with moderate to marked nuclear pleomorphism, arranged in herringbone, interlacing, or storiform patterns. Although most MCs are triple-negative,^3)^ they have low response rates to chemotherapy and a poor prognosis even among TNBCs,^4,5)^ highlighting the need for better treatment strategies. In recent years, the efficacy of ICIs in TNBC has been demonstrated,^6,7)^ leading to their clinical use in the perioperative setting. However, reports of ICI use in MC are scarce. Here, we report a case of SpCC in which preoperative chemotherapy combined with an ICI resulted in minimal therapeutic benefit. We subsequently performed a detailed analysis of the tumor immune microenvironment and explored potential factors contributing to the poor treatment response.

## CASE PRESENTATION

A woman with a 10-year history of a left breast mass re-presented because of subjective enlargement. Ten years earlier, the lesion measured 3 cm, and a core needle biopsy had been interpreted as fibroadenoma. However, as the patient discontinued follow-up on her own, no subsequent follow-up examinations were performed. On current assessment, it had enlarged to 7 × 4 × 4 cm, and repeat core needle biopsy revealed SpCC. IHC showed that the tumor cells were negative for estrogen receptor, progesterone receptor, and human epidermal growth factor receptor 2, confirming TNBC, and the Ki-67 labeling index was 70%–80%. It should be noted that the biopsy specimen obtained 10 years earlier was retrieved and re-examined, confirming the diagnosis of fibroadenoma, with no evidence of SpCC.

**Fig. 1:**
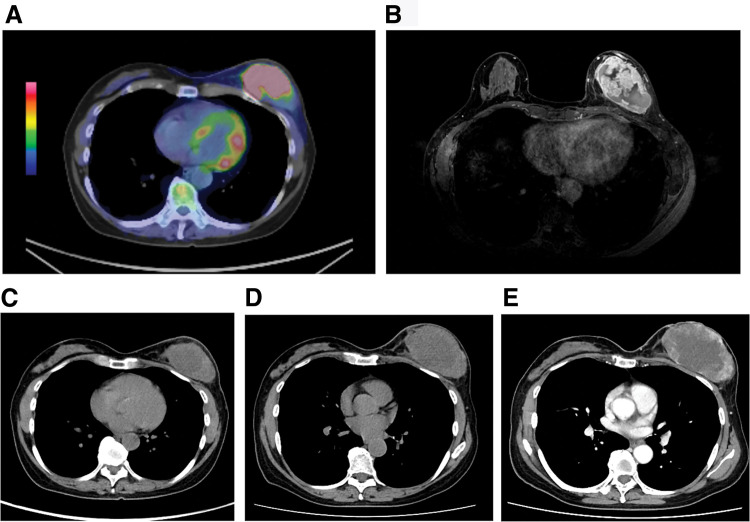
Serial radiological images before and during neoadjuvant chemo-immunotherapy. (**A**) PET/CT at presentation demonstrates a hypermetabolic left breast mass without evidence of distant metastasis. (**B**) Contrast-enhanced breast MRI at diagnosis reveals a well-defined enhancing lesion measuring approximately 7 cm, corresponding to the PET/CT findings. (**C**) CT at presentation demonstrates a left breast mass measuring approximately 7 cm. (**D**) CT prior to the initiation of chemo-immunotherapy shows interval enlargement to approximately 8.5 cm. (**E**) CT after 2 cycles of chemo-immunotherapy demonstrates further tumor enlargement to approximately 10 cm with ulceration, consistent with clinical progressive disease.

At presentation, PET/CT demonstrated a hypermetabolic left breast mass (**Fig. 1A**) and an enlarged ipsilateral axillary lymph node with increased uptake of FDG. Although fine-needle aspiration cytology of the enlarged node was negative, the pattern of FDG uptake and morphologic findings on PET/CT were highly suggestive of nodal involvement; therefore, the axilla was clinically regarded as node-positive (cN+). Contrast-enhanced breast MRI confirmed a well-defined enhancing lesion measuring approximately 7 cm (**Fig. 1B**). Based on these findings, the clinical stage was cT3 cN1 cM0, corresponding to cStage IIIA.

Given the diagnosis of TNBC, neoadjuvant chemo-immunotherapy was planned in accordance with the KEYNOTE-522 regimen. The planned regimen consisted of pembrolizumab 200 mg every 3 weeks combined with weekly paclitaxel (80 mg/m^2^) and carboplatin (area under the concentration–time curve 1.5) for 12 weeks, followed by epirubicin (90 mg/m^2^) plus cyclophosphamide (600 mg/m^2^) every 3 weeks for 4 cycles, with pembrolizumab continued throughout. To evaluate temporal changes in tumor size, serial CT scans were reviewed. CT at presentation demonstrated a left breast mass measuring approximately 7 cm (**Fig. 1C**). A subsequent CT performed before initiation of chemo-immunotherapy showed interval enlargement to approximately 8.5 cm (**Fig. 1D**). In addition, CT obtained after 2 cycles of chemo-immunotherapy demonstrated further enlargement to approximately 10 cm with ulceration, consistent with clinical progressive disease (**Fig. 1E**).

Neoadjuvant chemo-immunotherapy was initiated; however, after the second cycle, the tumor ulcerated and protruded through the prior punch-biopsy site, further supporting disease progression. Based on the clinical course and imaging findings, the treatment strategy was changed to surgical management, and the patient underwent a left total mastectomy with axillary lymph node dissection. No immune-related adverse events were observed during the clinical course.

### Pathological analysis of surgically resected specimen

**Fig. 2:**
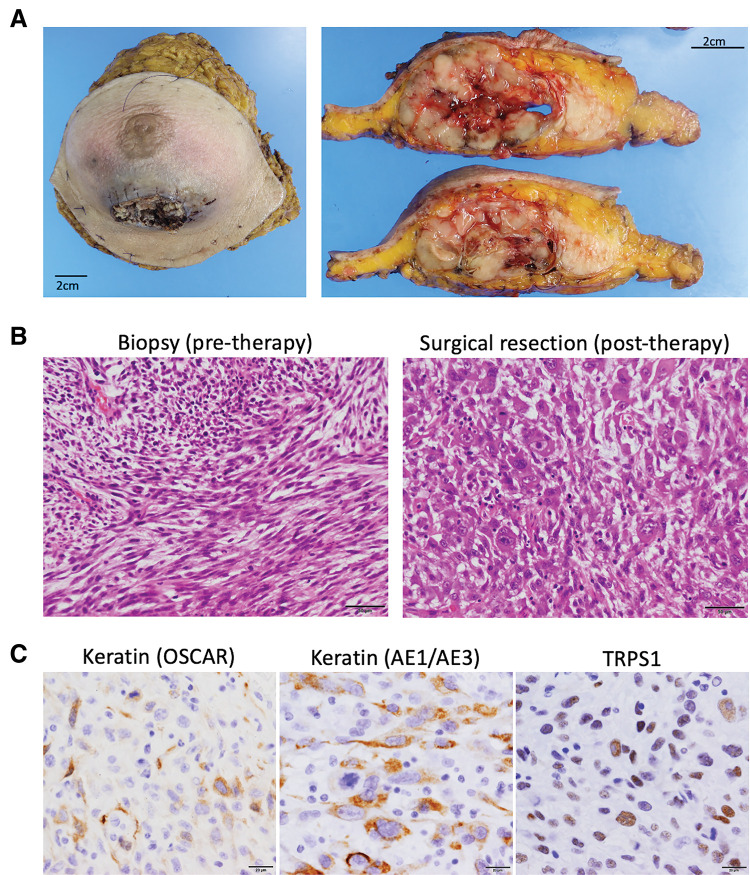
Pathological examination for diagnosis. (**A**) Gross observations of surgically resected breast cancer indicate a whitish nodular lesion with skin ulcer and necrosis. (**B**) Hematoxylin and eosin staining of breast cancer shows the proliferation of atypical spindle tumor cells. (**C**) IHC of keratin (clone OSCAR and clone AE1/AE3) and TRPS1 indicate that tumor cells are positive for keratin and TRPS1. IHC, immunohistochemistry

An ulcer measuring 5 × 3.5 cm was present, and a 10.1 × 6.8-cm mass was identified on the largest cut surface (**Fig. 2A**). Hematoxylin and eosin staining of the biopsy and surgically resected specimens is shown in **Fig. 2B**. The lesion comprised intersecting fascicles of atypical spindle-shaped cells with infiltrative growth and areas of necrosis. Mitotic figures were readily identified. Across the examined sections, no definite glandular structures or nested architectures were observed. The atypical spindle cells showed focal positivity for TRPS1 (a breast carcinoma marker), pan-cytokeratin (AE1/AE3), and cytokeratin (OSCAR) (**Fig. 2C**). These findings were consistent with SpCC. The histologic grade was grade III (tubule/structural formation 3, nuclear pleomorphism 3, mitotic score 2). The tumor directly invaded the overlying skin with ulceration (pT4b). Necrosis involved approximately 30% of the resected specimen and was considered secondary to hypoxia due to rapid tumor growth rather than an immune-mediated effect, as only scant lymphocytic infiltration was observed within the necrotic areas. Accordingly, the histologic treatment effect was judged to be minimal, indicating resistance to chemo-immunotherapy. No definite lymphovascular invasion was identified, and all surgical margins were negative. No lymph node metastasis was identified, and the pathological stage was ypT4b ypN0, corresponding to ypStage IIIB.

### Additional pathological analysis

**Fig. 3:**
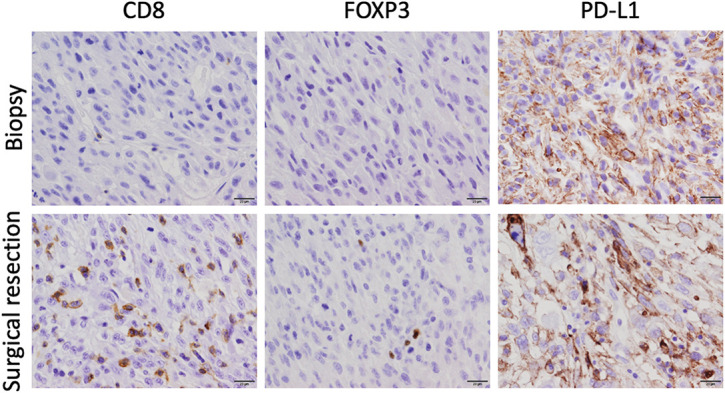
Additional examination of lymphocytes and PD-L1 expression. CD8-positive CTLs and FOXP3-positive Tregs are not seen in the biopsy sample but are observed in the resected sample. PD-L1 expression is high in both the biopsy and resected samples. CTL, cytotoxic T lymphocyte; PD-L1, programmed death-ligand 1; Treg, regulatory T cell

Next, we evaluated the tumor immune microenvironment and immune-related molecules, including PD-L1 and antigen presentation, by IHC (**Fig. 3**). Only small numbers of CD8+ CTLs and FOXP3+ Tregs were detected in the biopsy specimen, and the level of sTILs, assessed according to the International TIL Working Group guidelines, was 10%.^8)^ These immune cells were markedly increased in the surgically resected specimen. PD-L1 was strongly expressed in both tumor and immune cells in the biopsy and surgical specimens.

**Fig. 4:**
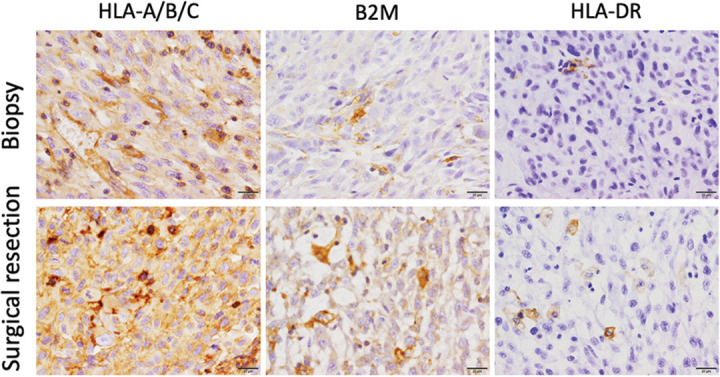
Additional examination of antigen presentation–related molecules. HLA-A/B/C (class I) is downregulated in cancer cells, but strongly positive in immune cells. B2M is negative or weakly positive in cancer cells. HLA-DR (class II) is negative in cancer cells. B2M, β2-microglobulin; HLA, human leukocyte antigen

Because downregulation of HLA antigens in tumor cells is considered to contribute to resistance to immunotherapy, we also assessed HLA-A/B/C, B2M, and HLA-DR. HLA-A/B/C and B2M were strongly positive in nucleated cells but only weakly positive in tumor cells (**Fig. 4**), indicating downregulation of HLA-A/B/C and B2M in tumor cells in both the biopsy and surgical specimens. HLA-DR was strongly positive in antigen-presenting cells, such as macrophages, but was negative in tumor cells in both specimens.

**Fig. 5:**
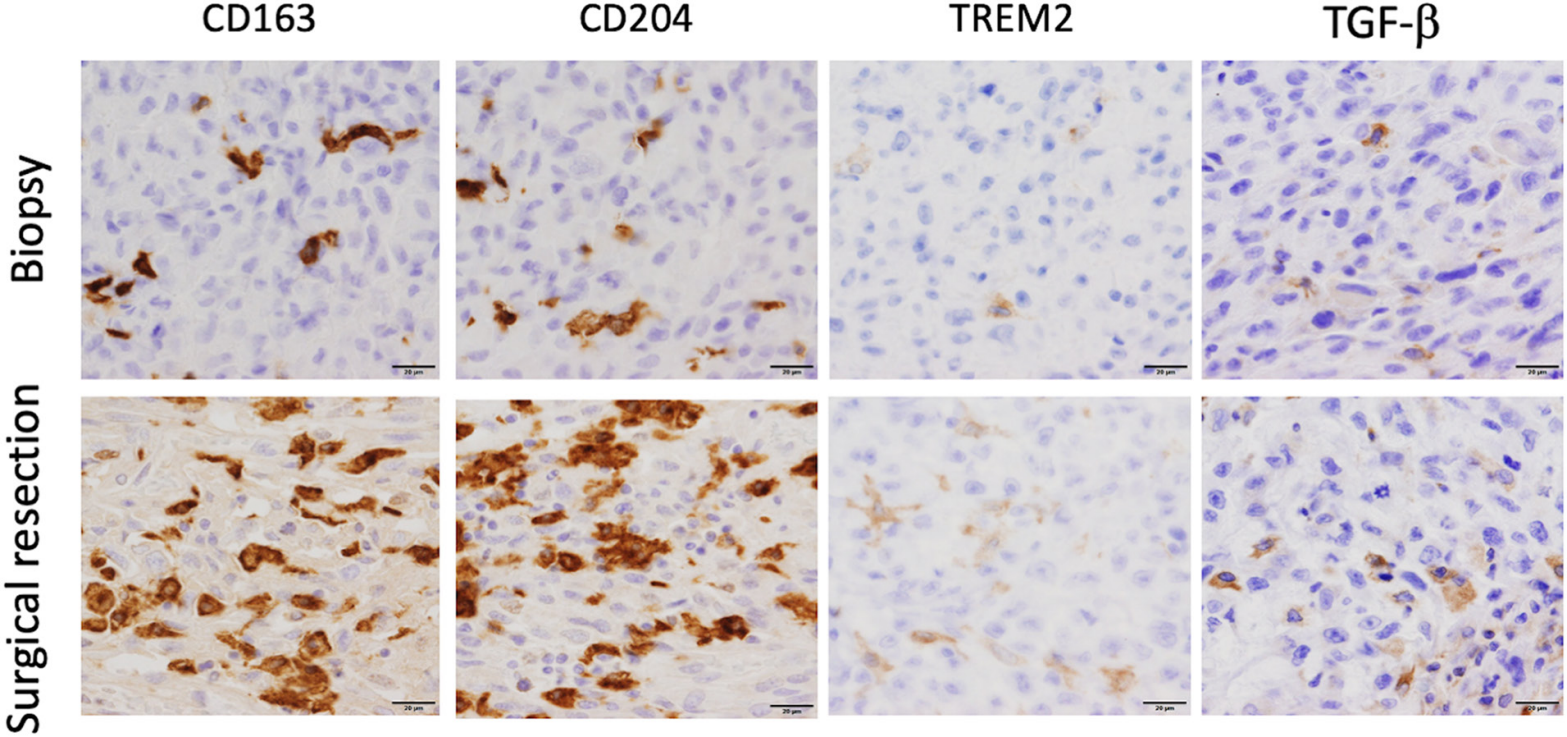
Additional examination of macrophage-related molecules. CD163 and CD204 are strongly positive in TAMs, and TREM2 and TGF-β are weakly positive in TAMs. TAMs are increased in the resected sample. TAM, tumor-associated macrophage; TGF-β, transforming growth factor-β; TREM2, triggering receptor expressed on myeloid cells 2

Macrophages infiltrating tumor tissues, known as TAMs, are thought to contribute to immunosuppression and resistance to chemo- and immunotherapy. CD163-, CD204-, and TREM2-positive TAMs were present in both the biopsy and surgical specimens, but were significantly increased in the surgical specimen (**Fig. 5**). TGF-β, a well-known protumor factor associated with chemoresistance and immune evasion, was negative in tumor cells but positive in infiltrating immune cells that morphologically appeared to be TAMs (**Fig. 5**).

**Fig. 6:**
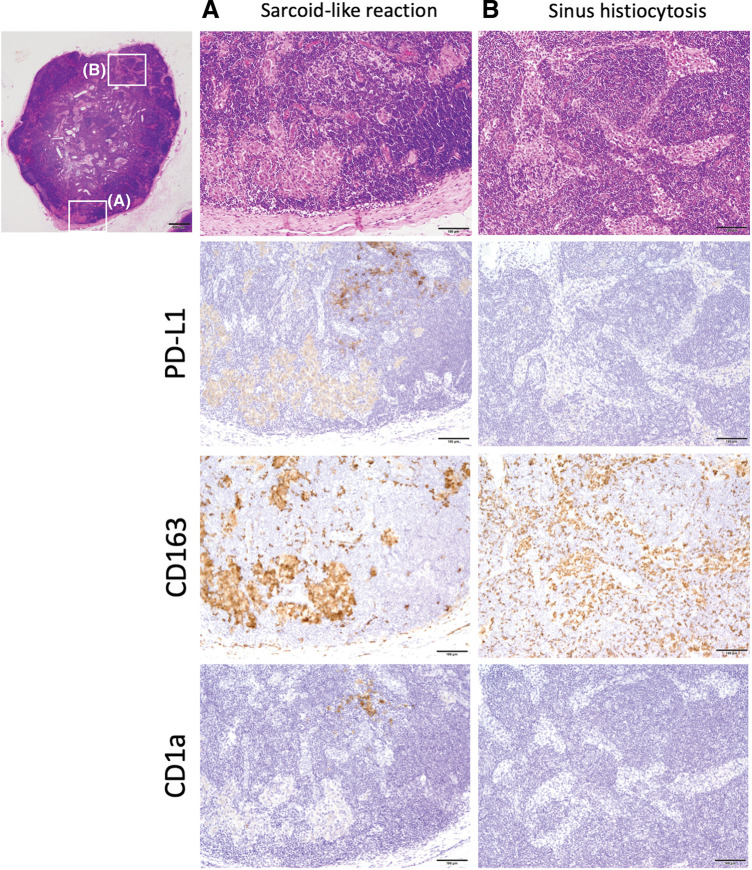
Additional examination of a resected nonmetastatic axillary lymph node. The area (**A**) in the upper left panel indicates sarcoid-like reaction, and the area (**B**) indicates sinus histiocytosis. (**A**) Sarcoid-like reaction and (**B**) sinus histiocytosis are observed. IHC of PD-L1, CD163, and CD1a was performed. IHC, immunohistochemistry; PD-L1, programmed death-ligand 1

Lymph nodes are critical lymphoid organs involved in antitumor immune responses. Therefore, we also examined PD-L1 expression in nonmetastatic regional axillary lymph nodes resected together with the breast tumor, along with CD163 and CD1a staining (**Fig. 6**). Sinus histiocytosis and a sarcoid-like reaction were observed. Strong PD-L1 expression was detected in the medullary area, where numerous CD1a-positive dendritic cells were present. PD-L1 showed weak positivity in CD163-positive macrophages within the sarcoid-like reaction, whereas PD-L1 was negative in macrophages associated with sinus histiocytosis.

## DISCUSSION

This case describes a breast mass with a 10-year history. A core-needle biopsy performed 10 years earlier showed fibroadenoma, whereas the current biopsy revealed SpCC. As no follow-up was performed during this interval, the radiological course remains unknown. In general, carcinomas associated with fibroadenoma are more commonly noninvasive lesions, such as ductal carcinoma in situ or lobular carcinoma in situ, whereas invasive carcinoma is rare and special histological types are considered extremely exceptional.^9)^ Taken together, the present SpCC is more likely to have arisen independently of the fibroadenoma.

In general, SpCC is resistant to ICI-based therapy^10)^; however, there has been a report of a remarkable response,^11)^ and the identification of predictive biomarkers is warranted. In the present case, HLA class I and B2M were markedly downregulated in tumor cells both before and after immunotherapy. HLA-DR was negative in tumor cells. It is well known that HLA class I downregulation and ectopic HLA-DR expression in tumor cells are associated with resistance and sensitivity, respectively, to ICI therapy in melanoma.^12)^ Furthermore, downregulation of B2M has also been suggested to be associated with resistance to ICI therapy in lung cancer.^13)^ Tumor cells frequently downregulate HLA class I to evade CD8+ T-cell recognition. Proposed mechanisms include mutations or loss of heterozygosity at HLA loci, B2M, or components of the antigen-processing machinery (e.g., TAP1/2, tapasin, endoplasmic reticulum aminopeptidase), which impair peptide loading and destabilize surface HLA expression.^14)^ Transcriptional and epigenetic suppression (e.g., promoter methylation, histone modifications) can also reduce HLA expression.^15)^ Inhibition of DNA methyltransferases upregulates HLA class I expression in cancer cells and enhances antitumor immune responses in mouse models,^16)^ with similar observations reported in melanoma.^17)^ Although further studies are required, DNA methyltransferase inhibition may be a potential strategy to reverse immune escape in MC.

sTILs have been identified as predictors of response to neoadjuvant chemo-immunotherapy in metaplastic TNBC, with cases showing <60% sTILs in pretreatment biopsy specimens being associated with treatment resistance.^10)^ In the present case, the low sTIL level may also have contributed to the observed resistance to therapy. In the present case, immunotherapy was associated with increased CTL infiltration; however, the antitumor effect was minimal. TAMs and Tregs are well known to exert immunosuppressive effects through the production of multiple inhibitory mediators. CD163 and CD204 are widely used markers associated with protumor TAM phenotypes,^18)^ and increased densities of CD163- and CD204-positive TAMs have been linked to worse outcomes in breast cancer.^19,20)^ TREM2 has also been reported as a key molecule enriched in protumor TAMs,^21)^ and anti-TREM2 antibodies have been shown to enhance the efficacy of immunotherapy by depleting TAMs in preclinical models.^22)^ The results of a clinical trial of a humanized anti-TREM2 monoclonal antibody suggested potential enhancement of ICI-mediated antitumor immunity.^23)^ TGF-β is a major immunosuppressive cytokine produced by various cell types, including Tregs and M2 macrophages.^24)^ In this case, a subset of TAMs expressed TGF-β. Thus, the increase in immunosuppressive immune cells may also have contributed to ICI resistance.

Lymph node sinus macrophages can present antigens via cross-presentation,^25)^ and their activation status has been associated with antitumor immune responses in several cancers.^26)^ Sinus macrophages express PD-L1, which may mediate immunosuppression through PD-1 signaling.^27,28)^ In the present case, PD-L1 expression appeared to be stronger in dendritic cells than in sinus macrophages. Macrophages within the sarcoid-like reaction showed weak PD-L1 expression, whereas PD-L1 was not detected in macrophages associated with sinus histiocytosis. CD163 was positive in sinus macrophages in both the sarcoid-like reaction and sinus histiocytosis; however, CD163 staining was stronger in the sarcoid-like reaction, suggesting that CD163 intensity may help distinguish these patterns. Sarcoid-like reactions have been reported after ICI therapy in several cancers and may be associated with improved overall survival.^29)^ In the present case, despite the presence of a sarcoid-like reaction, the tumor was resistant to ICI therapy. Nevertheless, the increased CTL density suggested that antitumor immune responses were upregulated. Therefore, in the present case, loss of HLA expression may represent a critical mechanism of ICI resistance. Antigen processing and presentation is a critical process in antitumor immunity and may therefore be relevant not only to SpCC and MC but also more broadly to TNBC. However, data regarding the relationship between HLA class I expression, its associated components, and the efficacy of ICIs in breast cancer remain limited, and further accumulation of evidence is warranted.

## CONCLUSIONS

We reported a case of MC resistant to ICI therapy. Despite evidence of increased immune activation, HLA loss may have contributed to treatment resistance. Assessment of HLA antigen expression before ICI therapy may be useful for predicting the likelihood of response to ICIs.

## DECLARATIONS

### Availability of data and materials

The data that support the findings of this study are available from the corresponding author upon reasonable request.
